# Intravitreal gas injection for early persistent macular hole after primary pars plana vitrectomy

**DOI:** 10.1186/s12886-022-02599-1

**Published:** 2022-09-18

**Authors:** Ying-Yi Chen, Chung-May Yang

**Affiliations:** 1 Department of Ophthalmology, National Taiwan University Hospital, Taipei, Taiwan; 2 Department of Ophthalmology, Cathay General Hospital, Taipei, Taiwan; 3 Graduate Institute of Clinical Medicine, College of Medicine, National Taiwan University, Taipei, Taiwan; 4 College of Medicine, National Taiwan University, Taipei, Taiwan

**Keywords:** Diameter hole index, Intravitreal gas injection, Macular hole diameter, Macular hole index, Optical coherence tomography, Persistent macular hole, Tractional hole index

## Abstract

**Purpose:**

To report the clinical presentations and outcome of early intravitreal injection (IVI) of octafluoropropane (C_3_F_8_) for persistent macular holes (MH) after primary pars plana vitrectomy with the internal limiting membrane (ILM) peeling technique.

**Methods:**

Nineteen eyes of 18 patients with persistent MH after vitrectomy underwent intravitreal injection of C_3_F_8_ between 11 and 21 days after the initial surgery (intravitreal gas injection group). Another nine eyes with a persistent MH without additional IVI C_3_F_8_ were included (non-intravitreal gas injection group).

Best-corrected visual acuity (BCVA), optical coherence tomography (OCT) features including size and configuration of MH, and time duration between the 2 surgeries were compared between the MH closure and open groups. The closure rate of persistent MHs was compared between the intravitreal gas injection group and non-intravitreal gas injection group.

**Results:**

Twelve of 19 eyes (63%) achieved MH closure after 1 to 3 times IVI C_3_F_8_. The final BCVA after vitrectomy and IVI gas was significantly better in the MH closure group (*P* = .005). Nine of 12 patients (75%) in the MH closure group had a visual acuity improvement of more than 2 lines. Original MHs with smaller minimal diameter, higher macular hole index (MHI) and higher tractional hole index (THI); and persistent MHs with smaller minimal diameter, higher THI, and lower diameter hole index (DHI) showed higher MH closure rate. None of the persistent MHs closed in the non-intravitreal gas injection group (0/9 eyes).

**Conclusion:**

Early intravitreal injection of C_3_F_8_ can be a cost-effective first-line treatment for early persistent MHs after primary surgery, especially in eyes with favorable OCT features.

## Precis

Intravitreal injection of C_3_F_8_ within 3 weeks of vitrectomy can be a cost-effective treatment for early persistent macular holes (MH), especially in eyes with smaller original MH, smaller persistent MH, and favorable MH characteristics.

## Introduction

A macular hole (MH) is a full-thickness defect in the central fovea, which causes reduced central vision. The main pathogenetic mechanism of idiopathic MH is oblique traction by the cortical vitreous on the fovea [1, 2]. Pars plana vitrectomy (PPV) with internal limiting membrane (ILM) peeling has been the primary surgical procedure for a MH, with an MH closure rate of 87 to 100% [3–6]. However, if the MH fails to close, central vision remains poor. Various techniques have been used to treat a persistent MH. The choice for secondary operations dealing with a persistent MH ranged from the simpler office-based fluid–gas exchange to obtain a large gas bubble [7], repeated vitrectomy with enlargement of the ILM rhexis with or without MH edge manipulation [8, 9], to various tissue flaps [10–12] with or without autologous platelet concentrate [13, 14]. Either expansile gas or silicone oil was used during re-operation [15]. One study showed repeated vitrectomy with ILM flap or radial nerve fiber layer incisions showed slightly higher closure rate than repeated gas injection alone but the difference was not significant [16]. Depending on the clinical setting, reoperations can achieve a pooled anatomical closure rate of 78% (95% CI 71-84%), with > 2-line best-corrected visual acuity (BCVA) improvement in 58% of patients [17]. One critical factor affecting the surgical outcome of a persistent MH is its duration. Repeated vitrectomy with fluid–air exchange and perfluoropropane (C_3_F_8_) gas injection within 3 months of the primary surgery results in better anatomical closure rates than late intervention [18]. The change of MH size and MH index after primary surgery were also found as markers for prognostic guidance of persistent MH [16, 19].

As most techniques for a persistent MH apply a large bubble for tamponade to increase the MH interface contact angle over a greater range of eye positions, we postulated that an increased gas volume in the early postoperative period may facilitate hole edge apposition and glial cell proliferation and migration. In this study, we performed intravitreal C_3_F_8_ injection for early persistent macula holes after vitrectomy and analyzed the MH closure rate, visual outcomes, and optical coherence tomography (OCT) characteristics of MH.

## Materials and methods

This retrospective review included consecutive patients with a persistent MH after primary surgery who received an additional intravitreal injection of C_3_F_8_between postoperative days 11 and 21. The primary surgery was vitrectomy with ILM peeling performed by the senior author (CMY) at the National Taiwan University Hospital from November 2007 to August 2020. In the same period, consecutive patients with a persistent MH after primary surgery, performed by CMY and other experienced surgeons, without additional intravitreal injection of C3F8 were also reviewed for comparison of the closure rate of persistent macular holes. This study was approved by the Research Ethics Committee of the National Taiwan University Hospital (REC ID: 202011050RINC) and was conducted in accordance with the Declaration of Helsinki. We used the STROBE case–control reporting guidelines [20]. The patients or the public were not involved in in the design, or conduct, or reporting, or dissemination plans of our research.

Complete ophthalmic examination, including BCVA and OCT imaging, was performed before vitrectomy, before IVI C_3_F_8_, and after IVI C_3_F_8_. Visual acuity was measured with a Snellen chart and expressed as a logarithm of the minimal angle of resolution (log MAR) for further analysis. The spherical equivalent refractive error was obtained using auto-refraction (Auto-kerato-refractometer KR-8800, Topcon, Tokyo, Japan). High myopia was defined as axial length > 26 mm or refractive errors <  − 6 diopters. OCT was performed using one of the following 3 machines at our clinic: RTVue XR Avanti (Optovue, Fremont, CA), RTVue RT100 (Optovue), and Cirrus HD-OCT (Carl Zeiss Meditec, Dublin, CA, USA).

**Fig. 1:**
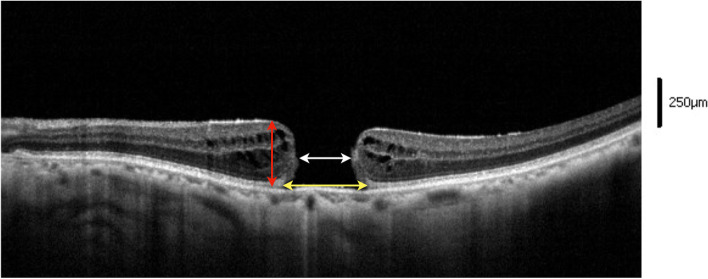
Measurement of the MH minimal diameter, MH basal diameter, and MH height. An 8-mm OCT scan of the left eye in case 11 obtained using Cirrus HD OCT (Carl Zeiss). The white arrow indicates MH minimal diameter (637 μm), and the yellow arrow indicates MH basal diameter (1011 μm). The red arrow indicates the height of MH margin (330 μm)

MH diameter was defined as the smallest linear distance of an MH in an area excluding the operculum, whereas basal diameter was defined as the distance of the base of an MH. MH height was defined as the thickness from the ILM to the retinal pigment epithelium at the margin of the hole measured on the horizontal section of OCT scans. The measurement of the above 3 MH diameters on the SD-OCT scan is demonstrated in Fig. 1. An examiner (YYC) blinded to participant data performed the measurement of the OCT scans. To demonstrate the reproducibility of the measured results, all OCT scans were measured twice on 2 days. The Bland–Altman method was used to determine the coefficients of repeatability as twice the standard deviation of the differences between 2 measurements. Three preoperative prognostic factors [21–23] including MH index (MHI), tractional hole index (THI), and diameter hole index (DHI) were calculated. MHI indicates the ratio of MH height to MH base diameter; THI indicates the ratio of maximum MH height to the minimum diameter of MH; DHI indicates the ratio of the minimum diameter of the hole to the diameter of the hole base.

### Primary surgical technique

Informed consent was obtained preoperatively. Under retrobulbar anesthesia, core vitrectomy was performed using a 23- or 25-gauge transconjunctival sutureless vitrectomy system (Alcon Laboratories, Fort Worth, TX, USA). The ILM was stained with 0.17% indocyanine green solution grasped with microforceps and peeled off at 360° for approximately 3 disc diameters around the MH. Air–fluid exchange was performed with a backflush needle, followed by intravitreal flush with 20 mL 15% C_3_F_8_ gas. The patients were instructed to take a prone position for 4 days after the operation.

### Secondary intravitreal gas injection technique

After the primary surgery, the patients were examined with indirect ophthalmoscopy daily during admission for 4 to 5 days. Then, the patients were followed up at outpatient clinic weekly within the first 3 weeks after primary surgery. When the gas bubble was gradually absorbed to less than 50% of vitreous cavity and allowed detection of a persistent macular hole, additional gas injection would be arranged between postoperative days 11 and 21 in the intravitreal gas injection group. Under topical anesthesia, an anterior chamber paracentesis of 0.15–0.25 ml was performed immediately prior to the intravitreal gas injection to prevent intraocular pressure spikes after injections. Then, 0.2–0.3 ml pure C_3_F_8_ gas was injected into the vitreous cavity through pars plana using a 27-gauge needle connected to a 3-ml syringe depending on the size of the residual gas within the vitreous cavity. The gas volume was increased in the eyes with longer axial length. Larger (0.3 ml) volume was used for those eyes containing less than 30% of gas. After the outpatient injection, the patient was instructed to take a prone position for 7 days until the next follow-up clinic. If the MH had not seal, but decreased in size by OCT, and the residual gas was less than 25% within 1 month postoperatively, another gas injection would be arranged. Maximal three times of weekly injection would be applied. Otherwise secondary vitrectomy with extension of ILM peeling/ILM flap technique would be adopted.

### Statistics analysis

Quantitative variables are presented as mean ± SD and categorical variables as frequencies and percentages. The Mann–Whitney U test was used for comparing continuous variables between 2 groups. The Wilcoxon signed-rank test was used to detect differences between variables before and after operation. For comparing categorical data, a chi-square test was used. Spearman correlation analysis was performed to evaluate the association between BCVA and MH measurements on OCT. *P* < 0.05 was considered statistically significant. All statistical analyses were performed using SPSS version 22. (SPSS, Chicago, IL, USA).

## Results

### Demographic data and clinical characteristics of the intravitreal gas injection group

Nineteen eyes of 18 patients (7 men and 11 women) underwent IVI C_3_F_8_ for early persistent MHs. Table 1 lists the demographic data, OCT features, number of gas injection, anatomical outcome, and visual acuity of the study patients. Patient age was 61.7 ± 14.2 years (range: 20–82 years). The mean refractive power of 19 eyes was − 1.93 diopters, and 3 eyes (15.8%) had high myopia. There were 15 phakic eyes and 4 pseudophakic eyes. The average follow-up duration was 75.4 ± 41.2 months (range: 10–132 months). On the basis of the preoperative SD-OCT, the mean minimal diameter of the MH was 498 ± 184 μm, whereas the mean basal diameter of the MH was 1202 ± 568 μm. The mean time interval between primary vitrectomy and first IVI was 15.0 ± 3.7 days, and the volume of IVI C_3_F_8_ was 0.24 ± 0.04 mL. Mean minimal diameter of the persistent MH measured 315 ± 168 μm by SD-OCT followed up within 3 weeks postoperatively.

**Table 1:** Demographic data, OCT features and the outcome in the intravitreal gas injection group

No	Age range	Gender	Refraction (D)	Diagnosis (stage)	Original MH diameter (μm)	Persistent MH diameter (μm)	Number of gas injection	Post-op MH status	Pre-PPV BCVA (logMAR)	Final BCVA (logMAR)
1	20 s	F	0.25	traumatic MH	444	208	1	closed	0.824	0.301
2	60 s	M	1.50	IMH(2)	278	-	1	closed	0.824	0.301
3	60 s	F	0.25	IMH(4)	468	146	1	closed	1.301	0.398
4	60 s	M	-3.00	IMH(4)	237	266	1	closed	0.824	0.523
5	70 s	M	3.00	IMH(3)	531	135	2	closed	1.301	0.398
6	40 s	F	-2.75	IMH(3)	475	152	2	closed	1.301	1.000
7	60 s	F	-7.50	MTM,MH	201	145	1	closed	0.903	0.301
8	30 s	F	-2.50^a^	MTM,MH	205	161	1	closed	0.398	0.097
9	50 s	F	-15.50	MTM,MH	341	171	3	closed	0.398	0.301
10	60 s	F	-2.00	IMH(4)	441	**371**	1	closed	0.824	0.222
11	70 s	M	-3.00	IMH(3)	637	279	1	closed	1.301	0.398
12	60 s	F	0.25	IMH(3)	**666**	345	1	closed	1.000	1.301
13	70 s	M	-0.50	IMH(3)	817	543	1	open	1.523	1.301
14	50 s	M	-0.75	IMH(3)	603	698	1	open	1.000	1.699
15	70 s	M	-1.00	IMH(4)	782	491	1	open	0.824	1.301
16	80 s	F	-4.00	IMH(3)	618	**270**	1	open	0.699	0.699
17	60 s	F	0.00	IMH(4)	657	371	2	open	1.301	1.699
18	60 s	F	-0.50	IMH(3)	553	350	1	open	1.301	1.495
19	60 s	M	1.00	IMH(3)	**509**	572	1	open	1.000	1.699

*No* Case number, *D* Diopter, *MH* Macular hole, *PPV* Pars plana vitrectomy, *BCVA* Best-corrected visual acuity, *logMAR* Logarithm of the minimum angle of resolution, *M* Male, *F* Female, *IMH* Idiopathic macular hole, *MTM* Myopic tractional maculopathy

^a^ Case 8 received LASIK previously, the axial length was 28.3 mm

### Anatomic outcome

**Fig. 2:**
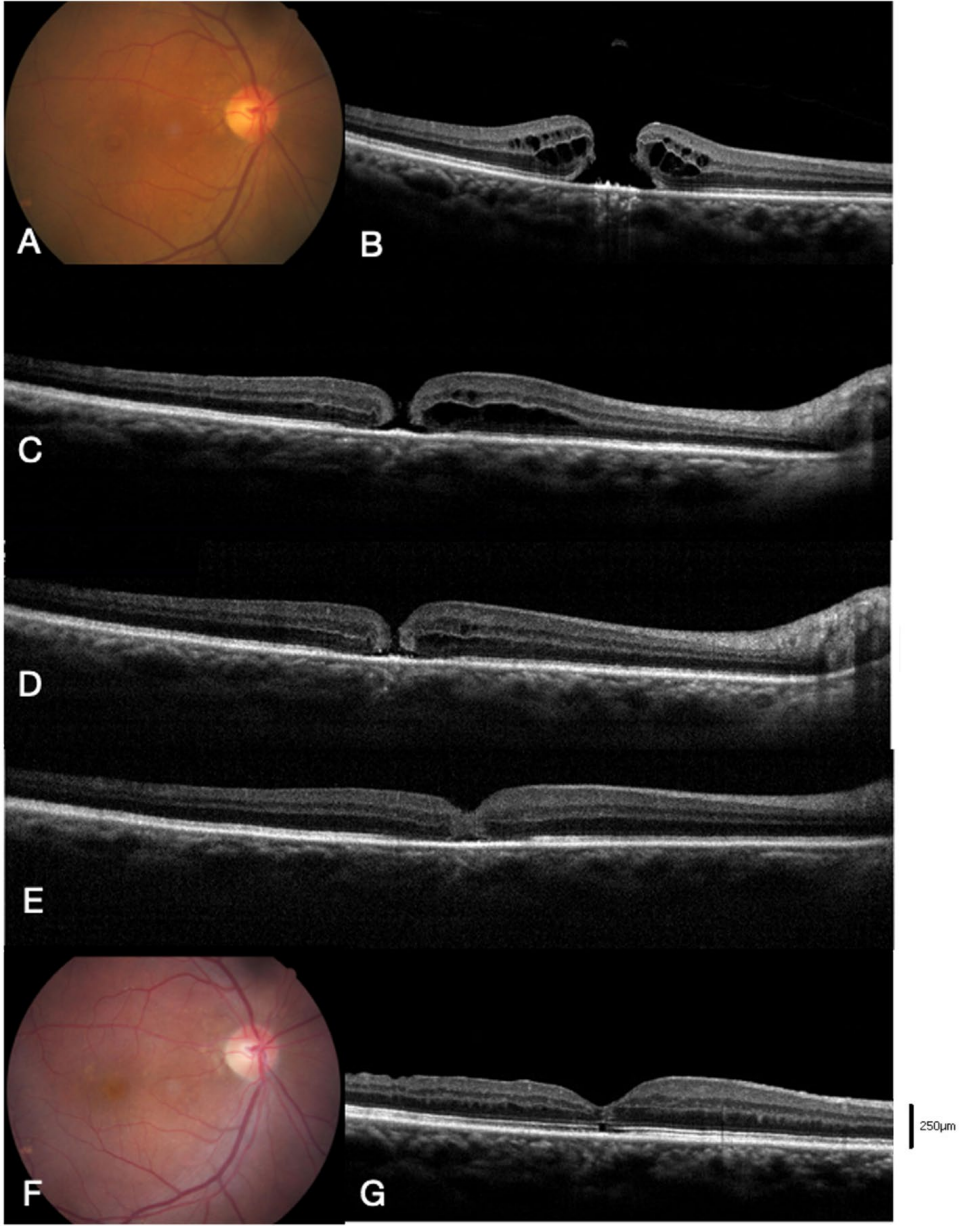
Case demonstration of case 5 in the MH closure group. **A**, **B** Preoperative color fundus and OCT image of the stage 3 MH with minimal diameter 531 μm. The MH base diameter was 1208 μm, and the height of MH margin was 390 μm. The patient received 23G pars plana vitrectomy, ILM peeling, IVI C_3_F_8_, and cataract surgery during the same operation. **C** At 12 days after the primary surgery, a 135 μm early persistent MH was noted by OCT image. IVI C_3_F_8_ 0.2 mL was administered, and the patient was kept in the prone position with face down. **D** At 20 days, MH size decreased to 89 μm. **E** At 2 months, MH closure and foveal gliosis were noted. **F**, **G** At 6 months, the foveal contour restored with focal interruption of the outer ellipsoid zone layers. The BCVA improved from 20/400 preoperatively to 20/50 6 months after surgery

**Fig. 3:**
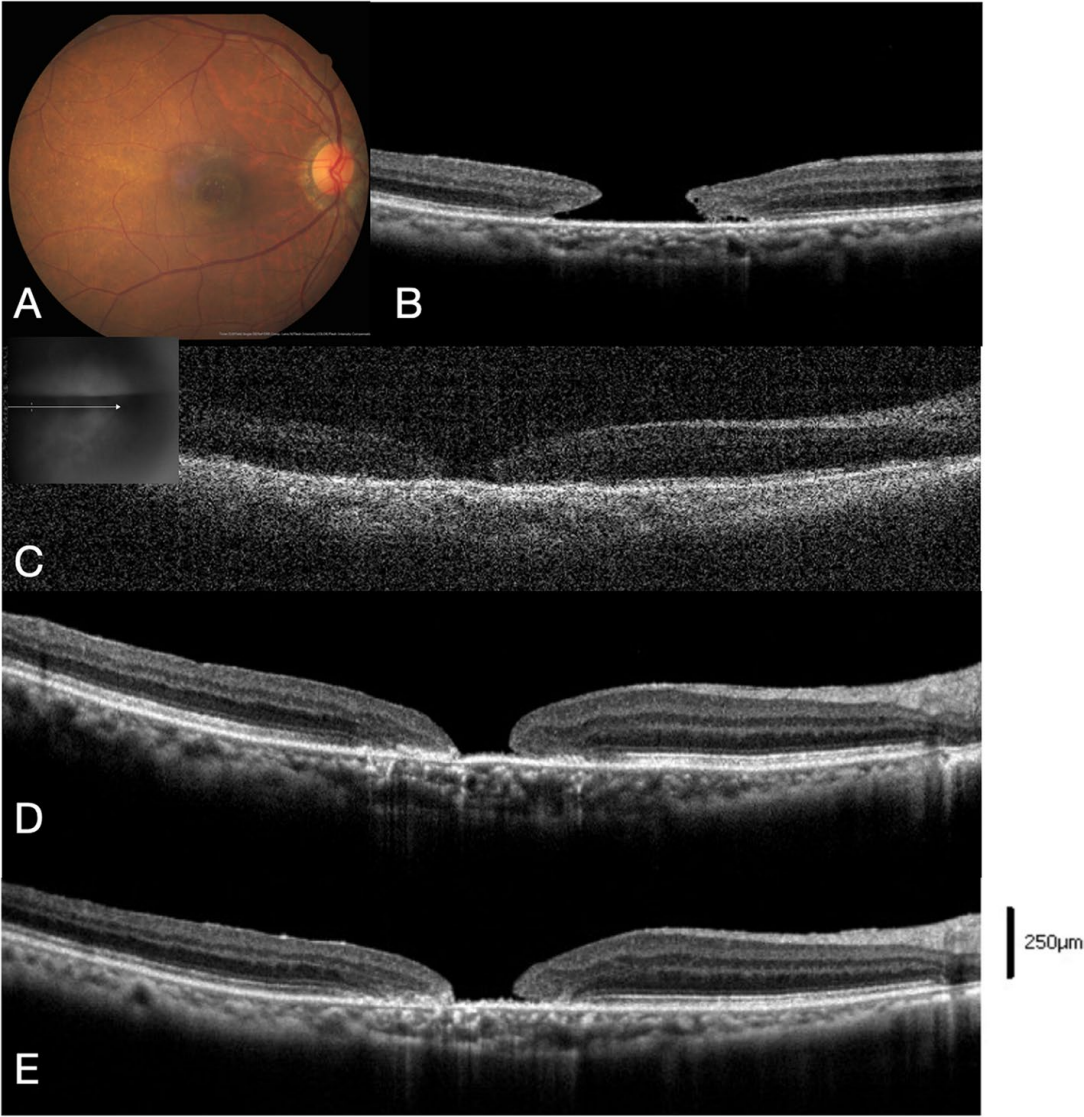
Case demonstration of case 17 in the MH open group. **A**, **B** Preoperative color fundus and OCT image of stage 4 MH (minimal diameter 657 μm, basal diameter 1285 μm). The margin of the hole was not elevated, and the margin height was 189 μm. **C** At 17 days after primary vitrectomy, ILM peeling, and IVI C_3_F_8_ 0.6 mL, a 371-μm early persistent MH was noted. Intravitreal injection of C_3_F_8_ 0.2 mL was performed. The image quality was influenced by the intravitreal gas–fluid interface. **D** At 1 month, the MH hole remained open. A second intravitreal injection of C_3_F_8_ 0.2 mL was added. **E** At 5 months, the MH showed no closure and the edge sealed

Twelve of 19 (63%) eyes achieved MH closure after IVI C_3_F_8_ (MH closure group). The other 7 eyes indicated persistent MHs after IVI C_3_F_8_ (MH open group). Three cases of the MH closure group (cases 5, 6, 9) and 1 case of the MH open group (case 17) underwent IVI C_3_F_8_ more than once. The representative cases of the 2 groups (cases 5 and 17) are presented in Figs. 2 and  3, respectively.

### Comparison of visual outcomes between the MH closure and MH open groups

For the mean age, sex ratio, refraction, time interval of additional IVI C_3_F_8_ after vitrectomy, BCVA before PPV and before IVI, no significant between-group differences were noted (Table 2). The best BCVA after primary vitrectomy and additional IVI with/without cataract extraction during follow-up was defined as the final BCVA. The final BCVA was significantly better in the MH closure group (log MAR 0.462 ± 0.344) than in the MH open group (log MAR 1.413 ± 0.362, *P* < 0.001). The final BCVA was significantly better than the previtrectomy BCVA in the MH closure group (*P* = 0.005). Nine of 12 patients (75%) in the MH closure group had a final BCVA improvement of ≥ 2 lines than the preoperative BCVA. The final BCVA was recorded at variable timing postoperatively considering cataract progression after vitrectomy. Three eyes (cases 2, 4, and 7) achieved best visual acuity after cataract extraction.

**Table 2:** Comparison of the clinical characteristics, visual acuity and OCT features of the macular hole in the macular hole closure group and the open group

	**MH closure group**	**MH open group**	***P* value**^**†**^
Number of eyes	12	7	
Age (y)	57.9 ± 15.4	68.3 ± 9.4	0.167
Sex (male/female)	4/8	4/3	0.311^‡^
Refraction (D)	-2.84 ± 5.05	-0.25 ± 0.79	0.711
first IVI time after PPV (day)	15.0 ± 7.3	17.1 ± 8.6	0.967
BCVA before PPV (logMAR)	0.933 ± 0.326	1.093 ± 0.293	0.384
BCVA before IVI C_3_F_8_ (logMAR)	1.200 ± 0.419	1.669 ± 0.598	0.413
Final BCVA (logMAR)	0.462 ± 0.344	1.413 ± 0.362	< 0.001***
BCVA improvement > 2 lines after IVI C_3_F_8_	9/12 (75%)	0/7 (0%)	
Time to final BCVA (month)	16.4 ± 32.8	5.0 ± 3.2	0.525
**OCT characteristics**			
Intraretinal cystoid edema	9/12	6/7	0.581^‡^
Original MH minimal diameter (μm)	410 ± 159	648 ± 114	0.005**
Original MH edge height (μm)	440 ± 137	329 ± 88	0.083
Original MH basal diameter (μm)	1218 ± 702	1174 ± 253	0.592
Macular hole index (MHI)	0.39 ± 0.20	0.29 ± 0.07	0.036*
Tractional hole Index (THI)	1.30 ± 1.09	0.52 ± 0.14	0.001**
Diameter hole Index (DHI)	0.43 ± 0.22	0.56 ± 0.06	0.432
Persistent MH minimal diameter (μm)	216 ± 85	471 ± 149	0.001**
Persistent MH edge height (μm)	352 ± 103	379 ± 69	0.681
Persistent MH basal diameter (μm)	728 ± 583	695 ± 296	0.669
Macular hole index (MHI)	0.57 ± 0.19	0.66 ± 0.24	0.606
Tractional hole Index (THI)	1.96 ± 0.83	0.91 ± 0.27	0.012*
Diameter hole Index (DHI)	0.41 ± 0.24	0.72 ± 0.14	0.010*
Postoperative IS/OS junction defect	7/12	7/7	

Final BCVA: the best BCVA noted after primary vitrectomy and additional IVI with/without cataract extraction during follow-up

MHI indicates the ratio of MH height to MH base diameter. THI indicates the ratio of maximum MH height to the minimum diameter of MH. DHI indicates the ratio of the minimum diameter of the hole to the diameter of the hole base

*MH* Macular hole, *y* Years old, *D* Diopter, *PPV* Pars plana vitrectomy, *BCVA* Best-corrected visual acuity, *logMAR* Logarithm of the minimum angle of resolution, *IVI* Intravitreal injection, *IS/OS* Inner segment/outer segment

^†^ Mann–Whitney U test

^‡^ Chi-square test

***P value<0.001; **P value <0.01 and *P value <0.05

### Comparison of OCT characteristics between MH closure and MH open group

Table 2 lists the OCT characteristics of MH, including presence of preoperative intraretinal cystoid edema, multiple MH measurement and calculated MH indices of original MH and persistent MH, and presence of postoperative inner segment/outer segment (IS/OS) defect. Further analysis of OCT features revealed a smaller original MH minimal diameter in the MH closure group (410 ± 159 μm) than the MH open group (648 ± 114 μm, *P* = 0.005). No significant difference was noted in the MH edge height or MH basal diameter between 2 groups. MHI and THI of the original MHs were significantly larger in the MH closure group (*P* = 0.036 and *P* < 0.001, respectively). Moreover, a significantly smaller minimal diameter of early persistent MH was found in the MH closure group (216 ± 85 μm) than the MH open group (471 ± 149 μm, *P* = 0.001). Significantly larger THI and smaller DHI of persistent MH were found in the MH closure group (*P* = 0.012 and *P* = 0.010, respectively).

With an original MH minimal diameter less than 509 μm, all the holes closed in our cases; with an original MH diameter greater than 666 μm, all the holes failed to close after PPV and IVI gas. Early persistent MH of minimal diameter less than 270 μm indicated 100% MH closure rate after IVI gas, whereas those with minimal diameter greater than 371 μm failed to close in our cases. (Table 1).

### Associated factors for visual outcome in MH closure group

In MH closure group, no significantly better final BCVA was found in the MH with intraretinal cystoid edema in the preoperative OCT (*P* = 0.100) or in eyes without IS/OS junction defect in postoperative OCT (*P* = 0.343). Cases 1, 5, 9, and 10 still achieved relatively good final BCVA, as the area of IS/OS junction defect was minimal after the foveal contour was restored (Fig. 2g). In the MH closure group, 5 eyes without IS/OS junction defect had final visual acuity better than log MAR 0.398 (Snellen 20/50) in this study. The other 7 eyes with MH closed but outer retinal layer defect not restored had variable final BCVA from log MAR 0.301(Snellen 20/40) to log MAR 1.301 (Snellen 20/400). The Spearman’s correlation coefficient of preoperative BCVA and final BCVA was 0.676 (*P* = 0.016), and that of preoperative MH minimal diameter and final BCVA was 0.635 (*P* = 0.027). Accordingly, the preoperative BCVA and MH minimal diameter were strongly associated with final BCVA in the MH closure group. No correlation between persistent MH measurements and the final BCVA was noted by nonparametric correlation (Spearman rank correlation) analysis.

### MH in high myopic eyes

A total of 3 high myopic cases (cases 7, 8, and 9) presented with better preoperative (*P* = 0.047) and postoperative BCVA (*P* = 0.023), smaller diameter of the original MH (*P* = 0.008), higher margin of original MH (*P* = 0.002), higher THI of persistent MH (*P* = 0.022), and lower DHI of persistent MH (*P* = 0.003) than those of the non-high myopic cases. All 3 high myopic cases had early persistent MH closed and final BCVA beyond 20/40.

### Complications

One case (case 17) developed acute angle closure attack during postoperative follow-up. Four cases (cases 4, 12, 17, and 18) underwent cataract surgery within 1 year after primary vitrectomy. Four of 7 eyes in the MH open group received repeated vitrectomy with ILM peeling (case 14 and 19) or lens capsular flap insertion (case 15 and 18). Although these MHs closed finally, outer retinal defect or foveal atrophy developed in the long term. Case 14 had rhegmatogenous retinal detachment 3 weeks after the second vitrectomy, necessitating a third operation.

### Comparison of the persistent MH closure rate between patients with and without additional C3F8 injection in early postoperative period

 Nine patients had prolonged face down positioning due to persistent MH detected after primary surgery (non-intravitreal gas injection group). Except further facedown for 7 to 10 days, no additional intravitreal injection of C_3_F_8_ was performed during postoperative day 11 to 21. The basic features of these 9 cases were shown in Table 3. The clinical characteristic and original MH diameter were similar in two groups. None of the persistent MHs closed in the non-intravitreal gas injection group (0/9) (Table 4). The persistent MHs became significantly larger during follow-up in this group (diameter of persistent macular holes before secondary operation: 460 ± 210 μm). Eight out of night patients received secondary PPV with enlargement of the ILM peeling area or using ILM flap insertion followed by expansile gas or silicone oil tamponade 13 weeks in average after the primary surgery. The patients who received early intravitreal gas injection achieved higher closure rate and better visual outcome than the patients with prolonged facedown only. (Table 4).

**Table 3:** Demographic data, OCT features and the outcome of the persistent macular hole in the non-intravitreal gas injection group

No	Age	Gender	Refract ion(D)	Diagnosis (stage)	Original MH diameter (μm)	Pre-PPV BCVA (logMAR)	Post-PPV BCVA (logMAR)	Persistent MH diameter before 2^nd^ PPV (μm)	Time to 2^nd^ PPV (week)	Final MH status
1	50 s	F	1.00	IMH(3)	511	1.301	1.301	504	5	closed
2	70 s	M	1.50	IMH(3)	238	1.301	1.699	426	21	closed
3	30 s	F	0.75	IMH(2)	176	-	2.000	414	12	closed
4	40 s	F	1.25	IMH(2)	840	0.523	1.301	862	7	closed
5	50 s	F	-1.50	MTM,MH	559	1.523	1.000	-	-	open
6	60 s	F	-2.50	LMH	232	0.699	1.523	306	10	closed
7	40 s	F	-11.25	IMH(3)	234	0.699	1.398	643	11	closed
8	60 s	M	0	IMH(3)	298	-	0.523	203	23	closed
9	60 s	M	-0.50	IMH(3)	283	-	1.301	320	15	closed

*No* Case number, *D* Diopter, *MH* Macular hole, *PPV* Pars plana vitrectomy, *BCVA* Best-corrected visual acuity, *logMAR* Logarithm of the minimum angle of resolution, *M* Male, *F* Female, *IMH* Idiopathic macular hole, *MTM* Myopic tractional maculopathy, *LMH* Lamellar macular hole

**Table 4:** Comparison of the demographic data, OCT features and the outcome of the persistent macular hole in the intravitreal gas injection group and the non-intravitreal gas injection group

Group	Number of eyes	Age	Gender (M:F)	Refraction (D)	Original MH diameter (μm)	Number of gas injection	Pre-PPV BCVA (logMAR)	Postoperative BCVA^a^ (logMAR)	MH Closure rate
Gas injection	19	61.8 ± 14.2	7:11	-1.9 ± 4.0	498 ± 184	1.3 ± 0.6	0.99 ± 0.32	0.81 ± 0.58	63% (12/19)
Non-gas injection	9	56.9 ± 11.4	3:6	-1.3 ± 4.0	375 ± 218	0	1.01 ± 0.42	1.44 ± 0.30	0% (0/9)
*P* value^†^		0.103	0.780^‡^	0.325	0.156		0.926	0.03	

*M* Male, *F* Female, *D* Diopter, *MH* Macular hole, *PPV* Pars plana vitrectomy, *BCVA* Best-corrected visual acuity, logMAR Logarithm of the minimum angle of resolution

^a^ In the intravitreal gas injection group, the BCVA after intravitreal gas injection with/without cataract extraction during follow-up was recorded. In the non-intravitreal gas injection group, the BCVA before secondary surgical intervention was recorded

^†^ Mann–Whitney U test

^‡^ Chi-square test

## Discussion

Various techniques have been adopted to treat persistent macular hole after surgery. Gas alone has been used instead of the more complicated vitrectomy. Either simple gas injection or fluid-gas exchange have been reported for this complication [7, 16–18, 24–27]. In this study, we found early supplementation of intravitreal C_3_F_8_ could achieve a MH closure in approximately 63% of cases with early persistent MH. In contrast, none of the cases with simple prolonged facedown but without additional gas injection obtained MH closure. Literature review of the technique similar to ours found that most contained small sample size and various success rate. Imai et al. [24] and Modi et al. [25] have documented 100% (5/5 eyes) and 75% (3/4 eyes) closure rates, respectively, using intravitreal C_3_F_8_ injection as a secondary intervention for persistent MH; a 20% (1/5 eyes) and 100% (7/7 eyes) closure rate was reported with intravitreal sulfur hexafluoride (SF_6_) injection [25, 26]. Our study included a larger sample size to validate the efficacy of intravitreal C_3_F_8_ injection alone as the first-line treatment of persistent MH. In addition to simple gas injection, outpatient fluid–gas exchange has also been used for cases with failed first MH surgeries. Rao et al. [7] reported an 89.6% (26/29 eyes) anatomic success with fluid–gas exchange by using 15% C_3_F_8_ or 20% SF_6_ 2–3 months after primary vitrectomy. Johnson et al. [27] achieved a 74% (17/23 eyes) success rate by fluid–gas exchange with 16–20% C_3_F_8_ within 1 week to 8 weeks after vitrectomy. Patel et al. [18] documented a 64% (16/25 eyes) rate of complete MH closure with fluid–air exchange and intravitreal C_3_F_8_ reinjection. As a comparison, the basal diameter in the MH open group of our study was 1174 ± 253 µm, with all 7 eyes > 900 μm and 71% of MH > 1000 μm. For Rao et al., MH in all cases (100%) was > 1000 μm [7]. For Patel et al., 78% of open MH after secondary fluid–air exchange had basal diameters of 400 to 1000 μm and 22% were > 1000 µm. [18]The success rate in our series was less than that of these 2 studies [7, 18]. However, simple gas injection may have the advantage of less complications, such as retinal trauma, hypotony, and lens damage.

Factors affecting the success rate of repeated surgery for persistent MH include duration of symptoms, surrounding cuff of fluid, MH with irregular/elevated edges, baseline MH diameter, minimum linear diameter (MLD) of persistent MH, decrease of MLD, and increase of MHI after primary surgery [5, 13, 14, 16, 19, 22]. In the current study, a smaller size of primary MH and persistent MH, higher MHI and THI of original MHs, as well as higher THI and lower DHI of persistent MHs were favorable OCT features for MH closure. Most of the cases had a decrease of MLD > 10% after the primary surgery (10/11 eyes in MH closure group; 5/7 eyes in MH open group, *P* = 0.280). The change of minimal MH diameter, MHI, THI and DHI after primary vitrectomy were not significantly different between the MH closure and the MH open groups.

To close a MH, vitreomacular traction should be released by vitrectomy and ILM peeling. Subsequent glial tissue growth was stimulated by ILM peeling and relied on the tamponade agent as migration template [28, 29]. Because inner retinal glial cell migration started from the base of the MH, smaller primary or persistent MH diameters had favorable anatomic outcomes. In our cohort, all eyes with original MH diameter < 509 μm or persistent MH diameter < 270 μm had complete MH closure after additional intravitreal C_3_F_8_ injection. For those eyes with original MH > 666 μm or persistent MH > 371 μm, the MH failed to close with intravitreal C_3_F_8_ injection alone. We suggest that repeated vitrectomy with air–fluid exchange or further ILM peeling/ILM flap transplantation and prolonged tamponade should be considered in such cases.

MHs with MHI > 0.5 or THI > 1.41 were reported to have a better prognosis after MH surgery by OCT studies [21–23]. The elevated edges of the MH may be free of adherence to underlying retinal pigment epithelium, which promotes the centripetal growth of glial tissue, thereby increasing the chance of MH closure. In our cases, significantly greater MHI and THI of original MHs and greater THI of persistent MHs were noted in the MH closure group. The average MH height was not significantly greater in the MH closure group, and we believed that the ratio of the MH height to MH diameter mattered more than the absolute MH height.

In our study, the BCVA significantly improved from log MAR 0.933 ± 0.326 to log MAR 0.462 ± 0.344 with MH closure and nine of 12 patients (75%) had a postoperative visual improvement of > 2 lines. In other studies using air–fluid exchange with gas tamponade, similar or worse postoperative visual improvement was reported. Johnson et al. [27] documented that 17 eyes (74%) with MH closure had their final visual acuity improved by ≥ 2 lines. Rao et al. [7] reported that BCVA for type 1 closure improved significantly from log MAR 1.66 ± 0.41 to 0.84 ± 0.41. Mean BCVA improved from log MAR 0.954 to log MAR 0.845, as reported by Patel et al. [18]

In the current study, we treated the patient less than 3 weeks after initial surgery. We reasoned that in the early postoperative period, the MH edge remained pliable without significant cystic change, hole enlarging, or epiretinal membrane formation. Thus, it may have the best opportunity to obtain MH closure with minor manipulation. In addition, 3 weeks after surgery, approximately 25% of the residual gas bubble was present in the intravitreal cavity; the addition of intravitreal gas with expansile property ensured a gas bubble of adequate volume to facilitate the approximation of the small foveal gap. This reasoning is supported by our result which showed prolonged facedown only without additional gas injection failed to obtain MH closure. Furthermore, earlier MH closure may improve functional results. In this study, repeated injections were performed for the MHs showing shrinking but not closing after the initial injection. We performed OCT every week to determine whether further injections are required; 3 of 4 cases had final MH closure after 2 to 3 injections. These results indicate that active injection in selected cases may increase the MH closure rate. We believe that the procedure performed in the early postoperative period may be an essential point for success, especially with the favored OCT features found in this study. This method was highly cost-effective and yielded clinically meaningful visual acuity improvement in our cases. We did not perform gas injection before 10 days because the macular status after the initial surgery could not be assessed adequately with ophthalmoscopy and OCT.

Our study is limited by its retrospective nature, lack of randomization, and small sample of patients who underwent repeated surgery with gas injection. Large original macular holes that underwent primary vitrectomy with inverted ILM-flap and large persistent macular holes that underwent secondary vitrectomy were not included in this study. Thus the ratio of original MH diameter < 500 µm was relatively high in our study subjects. Although the surgeries were performed by the same surgeon, the choice of optimal surgical methods may change over this long period of patient enrollment. Nonetheless, the macular hole closure rate of primary surgery was achieved in 91.2 and 95.3% of high myopia and control patients respectively [30]. We found patients with favorable MH configuration through an OCT scan may be most likely to benefit from additional gas injection. We also evidenced that intravitreal gas injection can offer comparable anatomic and visual outcomes with repeated vitrectomy in selected cases.

In conclusion, early intravitreal injection of C_3_F_8_ may be a cost-effective first-line treatment for early persistent MH after primary surgery. Smaller minimal diameter, higher MHI and THI of original MH, and smaller minimal diameter, higher THI, and lower DHI of persistent MH are favorable for MH closure. Future studies with a prospective design and a larger number of cases may be required to confirm our results.

## Data Availability

The datasets used and/or analyzed during the current study available from the corresponding author on reasonable request.
